# Crystal structure of pseudoguainolide

**DOI:** 10.1107/S2056989015002510

**Published:** 2015-02-11

**Authors:** Noureddine Beghidja, Samir Benayache, Fadila Benayache, David W. Knight, Benson M. Kariuki

**Affiliations:** a Unité de Recherche VARENBIOMOL, Constantine 1 University, Constantine 25000, Algeria; b School of Chemistry, Cardiff University, Main Building, Park Place, Cardiff CF10 3AT, Wales

**Keywords:** crystal structure, plant extract, *inula graveolens*

## Abstract

The lactone ring in the title mol­ecule, C_15_H_22_O_3_ (systematic name: 3,4a,8-tri­methyl­dodeca­hydro­azuleno[6,5-*b*]furan-2,5-dione), assumes an envelope conformation with the methine C atom adjacent to the the methine C atom carrying the methyl substituent being the flap atom. The other five-membered ring adopts a twisted conformation with the twist being about the methine–methyl­ene C—C bond. The seven-membered ring is based on a twisted boat conformation. No specific inter­actions are noted in the the crystal packing.

## Related literature   

For background to *inula graveolens*, see: Chiappini & Fardella (1980 ▸); Rustaiyan *et al.* (1987 ▸). For related structures, see: Herz *et al.* (1982 ▸); Schmidt *et al.* (1996 ▸); Wu *et al.* (2012 ▸); Billodeaux *et al.* (2014 ▸).
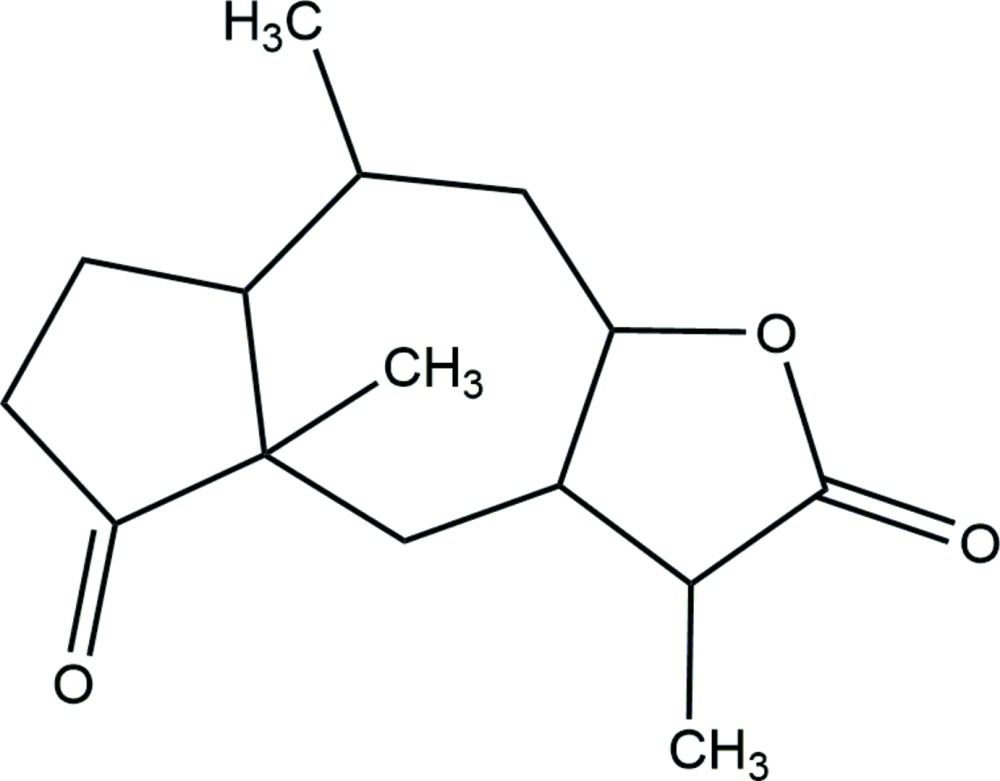


## Experimental   

### Crystal data  

C_15_H_22_O_3_
*M* *_r_* = 250.33
Orthorhombic, 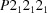

*a* = 7.4320 (3) Å
*b* = 11.9278 (3) Å
*c* = 15.3152 (6) Å
*V* = 1357.65 (8) Å^3^
*Z* = 4
Mo *K*α radiation
μ = 0.08 mm^−1^
*T* = 150 K
0.20 × 0.20 × 0.04 mm

### Data collection  

Nonius KappaCCD diffractometer
Absorption correction: multi-scan (*DENZO*/*SCALEPACK*; Otwinowski & Minor, 1997 ▸) *T*_min_ = 0.984, *T*_max_ = 0.997
9382 measured reflections
3098 independent reflections
2533 reflections with *I* > 2σ(*I*)
*R*_int_ = 0.041

### Refinement  

*R*[*F* ^2^ > 2σ(*F* ^2^)] = 0.047
*wR*(*F* ^2^) = 0.107
*S* = 1.08
3098 reflections
166 parameters
H-atom parameters constrained
Δρ_max_ = 0.15 e Å^−3^
Δρ_min_ = −0.16 e Å^−3^

### 

Data collection: *COLLECT* (Nonius, 2000 ▸); cell refinement: *SCALEPACK* (Otwinowski & Minor, 1997 ▸); data reduction: *DENZO* (Otwinowski & Minor, 1997 ▸) and *SCALEPACK*; program(s) used to solve structure: *SIR92* (Altomare *et al.*, 1999 ▸); program(s) used to refine structure: *SHELXL97* (Sheldrick, 2008 ▸); molecular graphics: *ORTEP-3 for Windows* (Farrugia, 2012 ▸); software used to prepare material for publication: *WinGX* (Farrugia, 2012 ▸).

## Supplementary Material

Crystal structure: contains datablock(s) I, New_Global_Publ_Block. DOI: 10.1107/S2056989015002510/tk5358sup1.cif

Structure factors: contains datablock(s) I. DOI: 10.1107/S2056989015002510/tk5358Isup2.hkl

Supporting information file. DOI: 10.1107/S2056989015002510/tk5358Isup3.cml

**Fig. 1.**
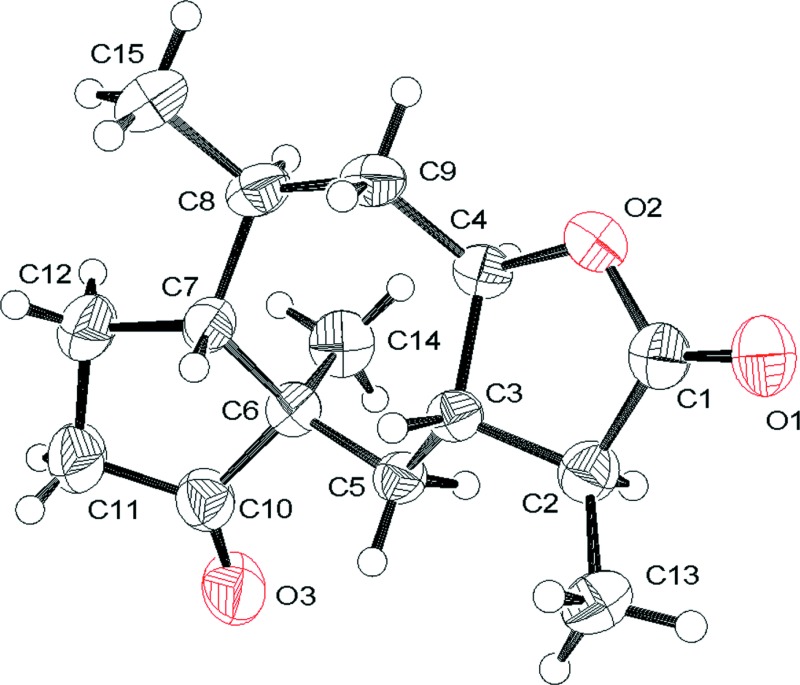
A molecule showing atom labels and 50% probability displacement ellipsoids for non-H atoms.

. DOI: 10.1107/S2056989015002510/tk5358fig1.tif
A mol­ecule showing atom labels and 50% probability displacement ellipsoids for non-H atoms.

CCDC reference: 1047797

Additional supporting information:  crystallographic information; 3D view; checkCIF report

## supplementary crystallographic information

### S1. Comment

*Inula graveolens* have consistently been the subject of research interest (Chiappini & Fardella, 1980; Rustaiyan *et al.*, 1987). Our interest is in the extracts from aerial parts of Algerian species such as stems, flowers and leaves. The asymmetric unit of the crystal structure consists of a single molecule (Fig. 1). In the molecule, the lactone ring assumes an envelope conformation. In the crystal structure, the planes of the lactone rings are approximately parallel. The molecules are arranged with the lactone rings stacked parallel to the *a* axis. Structures of some related compounds have been reported (Herz *et al.*, 1982; Schmidt *et al.*, 1996; Wu *et al.*, 2012; Billodeaux *et al.*, 2014).

### S2. Experimental

The air-dried aerial parts of *inula graveolens* (500 g) were extracted with acetone/Et_2_O (1:1) at room temperature. The solution was filtered off and concentrated under reduced pressure to give a pale-yellow gum (9 g). The gum was subjected to successive column chromatography (silica gel) and TLC (silica gel, PF254). Eleven fractions were obtained. Fraction 5 gave a material which crystallized as colourless crystals with a melting point of 152 *^o^*C.

### S3. Refinement

Carbon-bound H-atoms were placed in calculated positions (C—H = 0.96–0.98 Å) and were included in the refinement in the riding model approximation, with *U*_iso_(H) set to 1.2–1.5*U*_equiv_(C).

### Crystal data

**Table:**

C_15_H_22_O_3_	*D*_x_ = 1.225 Mg m^−^^3^
*M**_r_* = 250.33	Mo *K*α radiation, λ = 0.71073 Å
Orthorhombic, *P*2_1_2_1_2_1_	Cell parameters from 3098 reflections
*a* = 7.4320 (3) Å	θ = 3.1–27.5°
*b* = 11.9278 (3) Å	µ = 0.08 mm^−^^1^
*c* = 15.3152 (6) Å	*T* = 150 K
*V* = 1357.65 (8) Å^3^	Plate, colourless
*Z* = 4	0.20 × 0.20 × 0.04 mm
*F*(000) = 544	

### Data collection

**Table:**

Nonius KappaCCD diffractometer	3098 independent reflections
Radiation source: fine-focus sealed tube	2533 reflections with *I* > 2σ(*I*)
Graphite monochromator	*R*_int_ = 0.041
CCD slices, ω and φ scans	θ_max_ = 27.5°, θ_min_ = 3.1°
Absorption correction: multi-scan (*DENZO*/*SCALEPACK*; Otwinowski & Minor, 1997)	*h* = −9→9
*T*_min_ = 0.984, *T*_max_ = 0.997	*k* = −15→15
9382 measured reflections	*l* = −19→17

### Refinement

**Table:**

Refinement on *F*^2^	Primary atom site location: structure-invariant direct methods
Least-squares matrix: full	Secondary atom site location: difference Fourier map
*R*[*F*^2^ > 2σ(*F*^2^)] = 0.047	Hydrogen site location: inferred from neighbouring sites
*wR*(*F*^2^) = 0.107	H-atom parameters constrained
*S* = 1.08	*w* = 1/[σ^2^(*F*_o_^2^) + (0.0453*P*)^2^ + 0.2031*P*] where *P* = (*F*_o_^2^ + 2*F*_c_^2^)/3
3098 reflections	(Δ/σ)_max_ < 0.001
166 parameters	Δρ_max_ = 0.15 e Å^−^^3^
0 restraints	Δρ_min_ = −0.16 e Å^−^^3^

### Special details

**Table:**

Geometry. All s.u.'s (except the s.u. in the dihedral angle between two l.s. planes) are estimated using the full covariance matrix. The cell s.u.'s are taken into account individually in the estimation of s.u.'s in distances, angles and torsion angles; correlations between s.u.'s in cell parameters are only used when they are defined by crystal symmetry. An approximate (isotropic) treatment of cell s.u.'s is used for estimating s.u.'s involving l.s. planes.
Refinement. Refinement of *F*^2^ against ALL reflections. The weighted *R*-factor *wR* and goodness of fit *S* are based on *F*^2^, conventional *R*-factors *R* are based on *F*, with *F* set to zero for negative *F*^2^. The threshold expression of *F*^2^ > 2σ(*F*^2^) is used only for calculating *R*-factors(gt) *etc*. and is not relevant to the choice of reflections for refinement. *R*-factors based on *F*^2^ are statistically about twice as large as those based on *F*, and *R*- factors based on ALL data will be even larger.

### Fractional atomic coordinates and isotropic or equivalent isotropic displacement parameters (Å^2^)

**Table:**

	*x*	*y*	*z*	*U*_iso_*/*U*_eq_	
C1	0.8136 (2)	0.24699 (16)	0.52812 (12)	0.0383 (4)	
C2	0.8423 (3)	0.16380 (15)	0.45546 (12)	0.0407 (4)	
H2	0.9722	0.1503	0.4522	0.049*	
C3	0.7918 (2)	0.22980 (13)	0.37360 (11)	0.0318 (4)	
H3	0.6620	0.2220	0.3642	0.038*	
C4	0.8307 (2)	0.35109 (14)	0.40005 (11)	0.0338 (4)	
H4	0.9552	0.3703	0.3852	0.041*	
C5	0.8884 (2)	0.19024 (14)	0.29170 (12)	0.0346 (4)	
H5A	0.8386	0.1183	0.2748	0.041*	
H5B	1.0142	0.1785	0.3060	0.041*	
C6	0.8781 (2)	0.26974 (15)	0.21218 (12)	0.0371 (4)	
C7	0.7099 (3)	0.34762 (15)	0.20872 (12)	0.0388 (4)	
H7	0.6065	0.3039	0.2292	0.047*	
C8	0.7176 (3)	0.45618 (14)	0.26350 (13)	0.0429 (5)	
H8	0.8326	0.4932	0.2513	0.052*	
C9	0.7050 (3)	0.43722 (14)	0.36244 (12)	0.0396 (4)	
H9A	0.7276	0.5081	0.3914	0.048*	
H9B	0.5827	0.4152	0.3763	0.048*	
C10	0.8618 (3)	0.20169 (18)	0.12824 (14)	0.0495 (5)	
C11	0.7283 (4)	0.25562 (19)	0.06728 (14)	0.0630 (6)	
H11A	0.6211	0.2099	0.0617	0.076*	
H11B	0.7805	0.2665	0.0098	0.076*	
C12	0.6842 (3)	0.36830 (18)	0.10993 (14)	0.0583 (6)	
H12A	0.5613	0.3905	0.0973	0.070*	
H12B	0.7651	0.4264	0.0893	0.070*	
C13	0.7564 (3)	0.05319 (14)	0.47094 (13)	0.0453 (5)	
H13A	0.6282	0.0623	0.4737	0.068*	
H13B	0.7992	0.0226	0.5251	0.068*	
H13C	0.7864	0.0032	0.4240	0.068*	
C14	1.0567 (3)	0.33493 (19)	0.20250 (15)	0.0519 (5)	
H14A	1.0488	0.3846	0.1533	0.078*	
H14B	1.1536	0.2830	0.1937	0.078*	
H14C	1.0786	0.3777	0.2545	0.078*	
C15	0.5664 (3)	0.53701 (18)	0.23775 (17)	0.0650 (7)	
H15A	0.5670	0.6004	0.2764	0.098*	
H15B	0.4526	0.4992	0.2419	0.098*	
H15C	0.5847	0.5620	0.1788	0.098*	
O1	0.79726 (18)	0.22938 (12)	0.60507 (9)	0.0478 (3)	
O2	0.80792 (16)	0.35200 (10)	0.49559 (7)	0.0390 (3)	
O3	0.9465 (2)	0.11791 (15)	0.11224 (11)	0.0721 (5)	

### Atomic displacement parameters (Å^2^)

**Table:**

	*U*^11^	*U*^22^	*U*^33^	*U*^12^	*U*^13^	*U*^23^
C1	0.0265 (8)	0.0451 (10)	0.0433 (11)	−0.0014 (8)	−0.0003 (8)	0.0008 (8)
C2	0.0416 (10)	0.0404 (9)	0.0400 (10)	−0.0014 (8)	0.0015 (8)	0.0040 (8)
C3	0.0265 (8)	0.0298 (8)	0.0392 (9)	−0.0018 (7)	0.0004 (7)	0.0008 (7)
C4	0.0296 (9)	0.0335 (8)	0.0384 (9)	−0.0040 (7)	0.0040 (7)	−0.0020 (7)
C5	0.0314 (9)	0.0331 (9)	0.0393 (10)	0.0025 (7)	−0.0013 (8)	−0.0011 (7)
C6	0.0344 (9)	0.0381 (9)	0.0387 (10)	−0.0011 (8)	0.0020 (8)	0.0015 (8)
C7	0.0381 (9)	0.0357 (8)	0.0426 (10)	0.0007 (8)	−0.0058 (8)	0.0050 (8)
C8	0.0453 (11)	0.0301 (8)	0.0534 (12)	0.0008 (9)	0.0005 (9)	0.0064 (8)
C9	0.0373 (10)	0.0303 (8)	0.0512 (11)	−0.0012 (8)	0.0023 (9)	−0.0031 (7)
C10	0.0526 (12)	0.0532 (12)	0.0426 (12)	0.0015 (10)	−0.0019 (9)	−0.0027 (9)
C11	0.0807 (17)	0.0660 (14)	0.0424 (12)	0.0121 (14)	−0.0134 (12)	−0.0037 (10)
C12	0.0731 (15)	0.0544 (12)	0.0472 (12)	0.0092 (12)	−0.0115 (12)	0.0079 (9)
C13	0.0473 (11)	0.0383 (10)	0.0502 (12)	0.0076 (9)	0.0064 (9)	0.0056 (8)
C14	0.0441 (11)	0.0595 (12)	0.0522 (13)	−0.0096 (10)	0.0101 (10)	0.0073 (10)
C15	0.0772 (17)	0.0419 (11)	0.0761 (17)	0.0178 (12)	−0.0123 (14)	0.0068 (11)
O1	0.0432 (8)	0.0636 (8)	0.0367 (8)	−0.0003 (7)	0.0028 (6)	0.0023 (6)
O2	0.0380 (7)	0.0401 (7)	0.0388 (7)	−0.0038 (6)	0.0016 (6)	−0.0055 (5)
O3	0.0840 (12)	0.0763 (11)	0.0560 (10)	0.0307 (10)	−0.0134 (9)	−0.0248 (9)

### Geometric parameters (Å, º)

**Table:**

C1—O1	1.203 (2)	C8—C15	1.532 (3)
C1—O2	1.349 (2)	C8—C9	1.535 (3)
C1—C2	1.506 (3)	C8—H8	0.9800
C2—C13	1.485 (2)	C9—H9A	0.9700
C2—C3	1.527 (2)	C9—H9B	0.9700
C2—H2	0.9800	C10—O3	1.206 (2)
C3—C5	1.520 (2)	C10—C11	1.507 (3)
C3—C4	1.530 (2)	C11—C12	1.530 (3)
C3—H3	0.9800	C11—H11A	0.9700
C4—O2	1.473 (2)	C11—H11B	0.9700
C4—C9	1.503 (2)	C12—H12A	0.9700
C4—H4	0.9800	C12—H12B	0.9700
C5—C6	1.545 (3)	C13—H13A	0.9600
C5—H5A	0.9700	C13—H13B	0.9600
C5—H5B	0.9700	C13—H13C	0.9600
C6—C10	1.525 (3)	C14—H14A	0.9600
C6—C14	1.546 (3)	C14—H14B	0.9600
C6—C7	1.558 (3)	C14—H14C	0.9600
C7—C8	1.544 (3)	C15—H15A	0.9600
C7—C12	1.545 (3)	C15—H15B	0.9600
C7—H7	0.9800	C15—H15C	0.9600
			
O1—C1—O2	121.39 (17)	C9—C8—H8	107.9
O1—C1—C2	128.53 (18)	C7—C8—H8	107.9
O2—C1—C2	110.08 (15)	C4—C9—C8	116.18 (15)
C13—C2—C1	114.00 (15)	C4—C9—H9A	108.2
C13—C2—C3	118.92 (16)	C8—C9—H9A	108.2
C1—C2—C3	103.43 (14)	C4—C9—H9B	108.2
C13—C2—H2	106.6	C8—C9—H9B	108.2
C1—C2—H2	106.6	H9A—C9—H9B	107.4
C3—C2—H2	106.6	O3—C10—C11	124.8 (2)
C5—C3—C2	113.67 (14)	O3—C10—C6	124.83 (19)
C5—C3—C4	115.01 (14)	C11—C10—C6	110.31 (17)
C2—C3—C4	102.93 (13)	C10—C11—C12	104.58 (18)
C5—C3—H3	108.3	C10—C11—H11A	110.8
C2—C3—H3	108.3	C12—C11—H11A	110.8
C4—C3—H3	108.3	C10—C11—H11B	110.8
O2—C4—C9	107.71 (13)	C12—C11—H11B	110.8
O2—C4—C3	104.38 (13)	H11A—C11—H11B	108.9
C9—C4—C3	115.32 (15)	C11—C12—C7	104.55 (16)
O2—C4—H4	109.7	C11—C12—H12A	110.8
C9—C4—H4	109.7	C7—C12—H12A	110.8
C3—C4—H4	109.7	C11—C12—H12B	110.8
C3—C5—C6	115.87 (13)	C7—C12—H12B	110.8
C3—C5—H5A	108.3	H12A—C12—H12B	108.9
C6—C5—H5A	108.3	C2—C13—H13A	109.5
C3—C5—H5B	108.3	C2—C13—H13B	109.5
C6—C5—H5B	108.3	H13A—C13—H13B	109.5
H5A—C5—H5B	107.4	C2—C13—H13C	109.5
C10—C6—C5	109.98 (14)	H13A—C13—H13C	109.5
C10—C6—C14	104.76 (16)	H13B—C13—H13C	109.5
C5—C6—C14	109.96 (15)	C6—C14—H14A	109.5
C10—C6—C7	103.01 (15)	C6—C14—H14B	109.5
C5—C6—C7	115.63 (14)	H14A—C14—H14B	109.5
C14—C6—C7	112.70 (14)	C6—C14—H14C	109.5
C8—C7—C12	113.76 (15)	H14A—C14—H14C	109.5
C8—C7—C6	116.85 (15)	H14B—C14—H14C	109.5
C12—C7—C6	103.16 (15)	C8—C15—H15A	109.5
C8—C7—H7	107.5	C8—C15—H15B	109.5
C12—C7—H7	107.5	H15A—C15—H15B	109.5
C6—C7—H7	107.5	C8—C15—H15C	109.5
C15—C8—C9	107.59 (17)	H15A—C15—H15C	109.5
C15—C8—C7	111.13 (17)	H15B—C15—H15C	109.5
C9—C8—C7	114.24 (14)	C1—O2—C4	110.89 (13)
C15—C8—H8	107.9		
