# Supplementary figures and images for: Crystal structure of pseudoguainolide
Source: Acta Crystallogr E Crystallogr Commun. 2015 Feb 11;71(Pt 3):o162. doi: 10.1107/S2056989015002510 (PMC4350757; doi:10.1107/S2056989015002510)

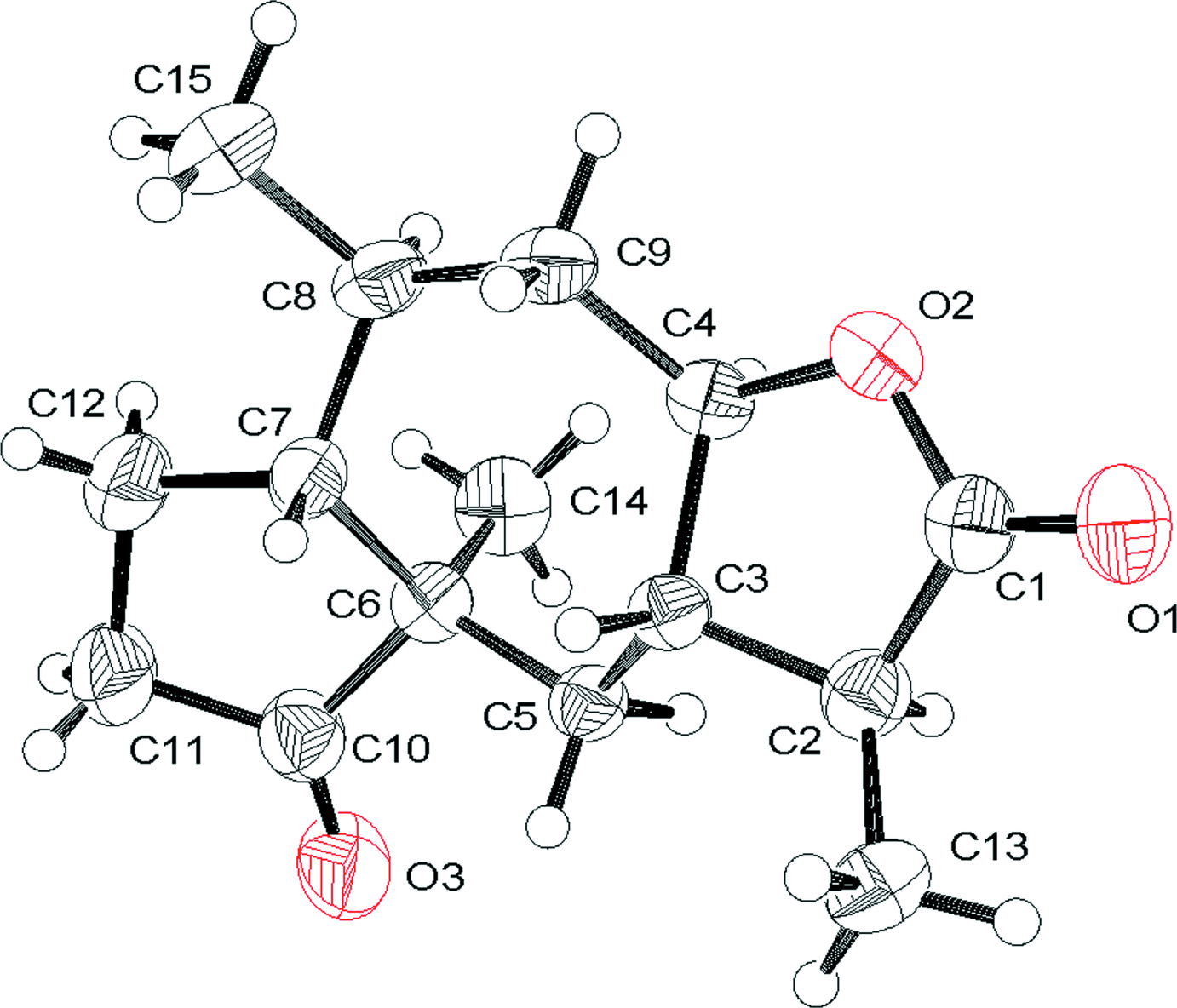

Supplement: Supplementary file 4 [file e-71-0o162-fig1.tif]
